# Fatal Melioidosis in Goats in Bangkok, Thailand

**DOI:** 10.4269/ajtmh.14-0115

**Published:** 2014-08-06

**Authors:** Walaiporn Tonpitak, Chulabha Sornklien, Mongkol Chawanit, Suvarin Pavasutthipaisit, Vanaporn Wuthiekanun, Viriya Hantrakun, Premjit Amornchai, Janjira Thaipadungpanit, Nicholas P. J. Day, Samuel Yingst, Sharon J. Peacock, Direk Limmathurotsakul

**Affiliations:** Department of Microbiology, Ruminant Clinic and Department of Pathology, Faculty of Veterinary Medicine, Mahanakorn University of Technology, Bangkok, Thailand; Mahidol-Oxford Tropical Medicine Research Unit, Department of Microbiology and Immunology and Department of Tropical Hygiene, Faculty of Tropical Medicine, Mahidol University, Bangkok, Thailand; Center for Clinical Vaccinology and Tropical Medicine, Nuffield Department of Clinical Medicine, University of Oxford, United Kingdom; Department of Epidemiology and Diseases Surveillance, Armed Force Research Institute of Medical Science, Bangkok, Thailand; Department of Medicine, University of Cambridge, Cambridge, United Kingdom

## Abstract

Bangkok, Thailand, is a city considered to be at low risk for melioidosis. We describe 10 goats that died of melioidosis in Bangkok. Half of them were born and reared in the city. Multilocus sequence typing ruled out an outbreak. This finding challenges the assumption that melioidosis is rarely acquired in central Thailand.

Melioidosis, an often fatal infectious disease for humans and animals, is caused by the Gram-negative bacillus and biothreat select agent *Burkholderia pseudomallei*.^1^ This organism is present in soil and water in melioidosis-endemic regions of the world, including much of Asia, northern Australia, regions of South America, some countries in Africa, and various Pacific and Indian Ocean islands.^1^ Most infections in humans and animals occur after skin inoculation, inhalation, or ingestion of the organism from the environment. A wide range of animal species are susceptible to melioidosis, including sheep, goats, swine, horses, cats, dogs, and non-human primates.^2^

We recently described the first report of culture-confirmed melioidosis in animals in Thailand, in which goats were the most frequently affected species.^3^ The regions where animal melioidosis were reported mapped to those areas where melioidosis is endemic in humans, including northeastern, eastern, southern, and western Thailand. To our knowledge, animal melioidosis has not been reported from central Thailand, and melioidosis is not considered to be endemic in humans in this area.^1^,^4^ Here, we describe 10 goats that died of melioidosis in Bangkok in central Thailand.

Study animals underwent necropsy as part of a routine service at the Veterinary Diagnostic Center, Mahanakorn University of Technology, Nong Chok District, Bangkok, Thailand. Organs with gross pathologic lesions were cultured on bovine blood agar and MacConkey agar. Presumptive *B. pseudomallei* colonies were confirmed by using conventional biochemical tests, multiplex polymerase chain reaction,^5^ and latex agglutination tests.^6^ A total of 72 goats and 367 other animals were necropsied during 2006–2012. Ten goats (14%) had at least one specimen that was culture positive for *B. pseudomallei*, and all other animals were culture negative for this species.

The ten goats were from six different farms (range = 1–4 goats/farm) located in two districts in Bangkok (Nong Chok and Khlong Sam Wa). The median age of affected goats was three years (range = 2–4 years), and nine (90%) were female. Common symptoms before death were pneumonia (n = 9), weakness (n = 4), anorexia (n = 2), neurologic symptoms (n = 2), and mastitis (n = 2). All cases had more than one organ involved, and multiple abscesses in the lung, liver, and spleen were common (Table 1). Milk was culture positive for two cases.

Histopathologic examination of the heart, lung, liver, spleen and kidney was conducted for four cases (cases 5, 7, 8, and 10). Acute necrotizing and granulomatous inflammation was found in the lung, liver, spleen and kidney in all four cases, and myocardial and endocardial hemorrhage was observed in three cases (cases 7, 8, and 10). Multinucleated giant cells were observed in the lung and spleen in one case (case 8) (Figure 1).

**Figure 1.**
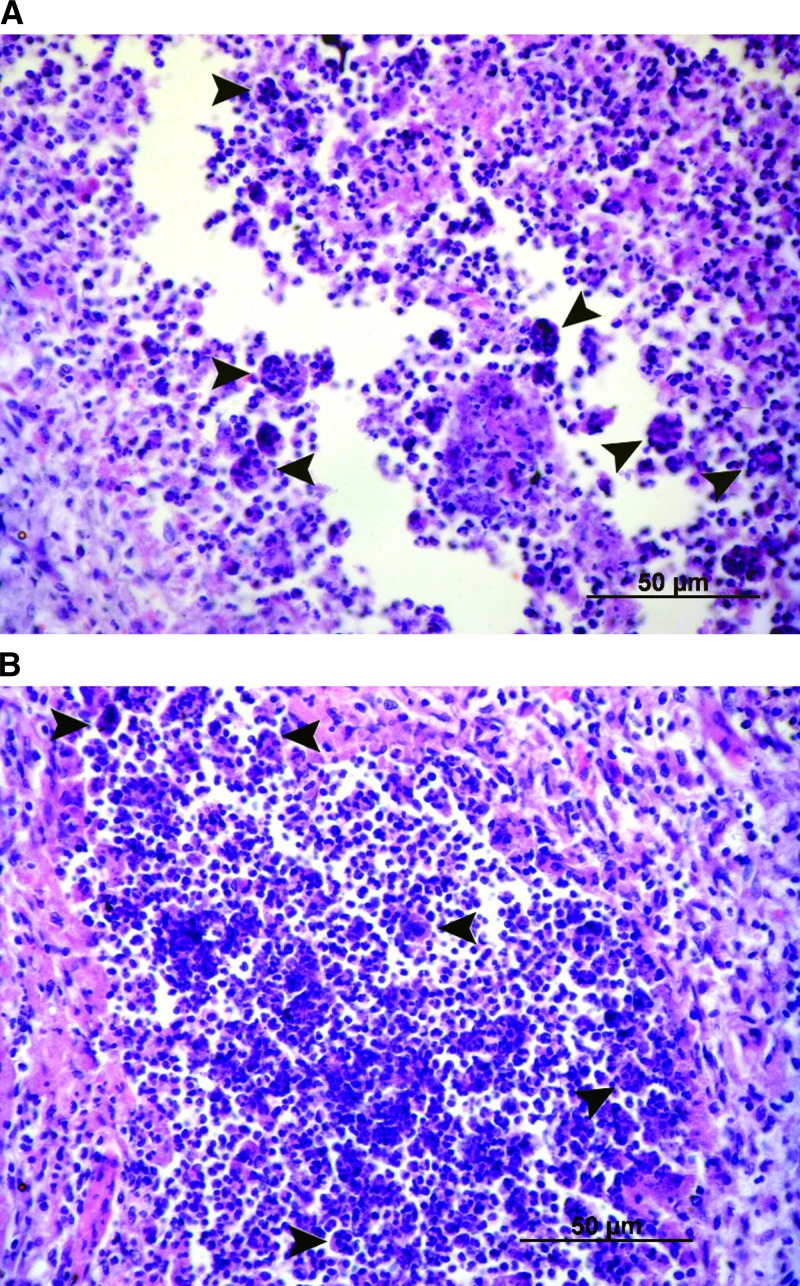
Hemotoxyin and eosin–stained tissue from lung (**A**) and kidney (**B**) of goats with melioidosis, showing multinucleated giant cells (**black arrows**) (original magnification ×400).

A total of 10 *B. pseudomallei* isolates from the 10 goats (1 isolate from each goat) were typed by multilocus sequence typing as described,^7^ which generated six sequence types (STs). Of these types, five STs (ST46, ST70, ST168, ST169, and ST188) have been identified in isolates originating in Southeast Asia (http://bpseudomallei.mlst.net), and the remaining ST (ST1089) was novel (Table 1). ST70, which has been reported to be the most abundant ST associated with clinical disease in Ubon Ratchathani in northeastern Thailand,^8^ was isolated from three goats from two farms (A and B). ST169, which was isolated from three of four goats from Farm D, has been isolated from clinical samples originating from Thailand, Vietnam, and Cambodia.

Five affected goats were born and raised in Bangkok and had no history of movement (Table 1). This finding raised the possibility that *B. pseudomallei* was endemic to the affected farms. In January 2013, an environmental survey was conducted at farm D, where four goats had died of melioidosis in 2010. Soil and water samples were collected and cultured as described.^9^,^10^ A total of 165 soil samples (10 grams/sample) and 40 water samples (1 L/sample) were collected, and none were culture positive for *B. pseudomallei.* The remaining five farms had been renovated, and environmental sampling was not possible.

We describe 10 cases of melioidosis in goats in Bangkok in central Thailand. Five goats were born and raised in the farms where they died, making it likely that they acquired melioidosis on these farms. Based on these findings, we propose that Bangkok may be an at-risk area for melioidosis. Bangkok is not considered to be endemic for melioidosis, and one explanation for this apparently changing pattern of epidemiology is the transportation of infected animals into Bangkok from known melioidosis-endemic areas.

This finding echoes the outbreak of melioidosis and dissemination of *B. pseudomallei* caused by imported animals that occurred in the Jardin des Plantes incident in Paris in 1975, in which a sustained outbreak of melioidosis in captive animals was believed to have originated from a panda imported from China.^11^ A number of animals were infected throughout France, and *B. pseudomallei* was subsequently detected in soil in many locations in the country at that time.^11^

In our study, three goats were purchased from other farms before death and two goats had an unknown history of origin. The other five goats who were born and raised in their farm may have acquired melioidosis from introduced animal(s). The presence of infected animals on Bangkok farms would be predicted to introduce *B. pseudomallei* into the environment, but this suggestion was not confirmed during a limited environmental survey performed on one farm.

Case demographics and bacterial genotypes show that goat melioidosis acquired in Bangkok was not linked to a single outbreak; 10 cases occurred on six farms over a seven-year period. Although four goats died of melioidosis at farm D in 2010, genotyping data showed that these deaths were caused by two genotypes (ST46 and ST169). The remaining six cases in the other five farms were caused by four STs. Nonetheless, it is possible that goats may have acquired *B. pseudomallei* from multiple recent introductions. This suggestion is supported by the fact that all three strains from farms A and B in 2006 were the same genotype (ST70), and that three of four strains from farm D in 2010 belonged to another single genotype (ST169).

It is also possible that goats may have acquired *B. pseudomallei* that was covertly present in the environment in Bangkok, rather than recently introduced. Three previous studies have evaluated the presence of *B. pseudomallei* in soil in central Thailand. A study by Finkelstein and others reported negative results for *B. pseudomallei*.^12^ Vuddhakul and others^13^ and Smith and others^14^ reported the presence of *B. pseudomallei*, but isolates were later identified as the highly related but non-pathogenic *Burkholderia thailandensis* (Smith and others, unpublished data). The presence of goat melioidosis in Bangkok, together with the report of melioidosis patients in central Thailand by Vuddhakul and others in 1999,^13^ raises the possibility that *B. pseudomallei* may be present in this region. Further studies are underway to evaluate the presence of *B. pseudomallei* across Bangkok and central Thailand.

There are no pathognomonic histopathologic findings for melioidosis. The findings in our cases are similar to those reported for samples from animals and humans with melioidosis, in which acute necrotizing and granulomatous inflammation were commonly observed.^2^,^15^,^16^ Multinucleated giant cells, which have been reported in human and goat melioidosis,^2^,^15^,^16^ were observed in only one of four cases examined in our study.

Melioidosis is difficult to diagnose and may be unrecognized because diagnostic confirmation relies on microbiologic culture and microbiologic expertise. *Burkholderia pseudomallei* is commonly dismissed as a culture contaminant, or may be misidentified as *Pseudomonas* spp. or other organisms by standard identification methods, including API 20NE and automated bacterial identification systems. Therefore, it is possible that the 10 fatal goat melioidosis cases reported from one Veterinary Diagnostic Center may represent the tip of the iceberg for animal melioidosis in Bangkok. Our findings suggest that melioidosis may be endemic to Bangkok in central Thailand. Considering the known potential for outbreaks of melioidosis in livestock, we suggest that melioidosis should be included in the animal disease control program in Thailand. In addition, mastitis and *B. pseudomallei*–contaminated goat milk is common in goat melioidosis,^3^,^16^ and we suggest that goat milk should be pasteurized before consumption in Thailand.

## Figures and Tables

**Table 1:** Characteristics of 10 goats that died of culture-confirmed melioidosis in Bangkok, Thailand, 2006–2010

Case no.	Year	Farm	Age, years	Sex	Origin	Presenting symptoms	Organs with gross pathology showing abscesses†	Organs with gross pathologic changes showing other abnormalities	Sequence type determined by MLST‡
1	2006	A	2	F	Born and raised in farm A	Lethargy, weakness, nasal discharge, dyspnea, and bloody mucoid diarrhea	**Lung, liver, spleen**	Aorta, adrenal gland, pleura, nasal cavity, mandibular LN*	70
2	2006	B	3	F	Unknown history	Anorexia, chronic pneumonia, nasal discharge, abdominal distension	Mandibular LN, **prefemoral LN**, spleen	−	70
3	2006	B	3	F	Unknown history	Anorexia, chronic pneumonia, nasal discharge	**Lung, kidney,** spleen	−	70
4	2008	C	4	F	Bought from other farms	High fever, anorexia, mastitis, hemiparesis, pneumonia	Mammary gland **(milk), retropharyngeal LN, pleural cavity**, spleen, brain	−	188
5	2010	D	3	F	Born and raised on farm D	Weakness, lethargy, pneumonia	**Lung, liver, spleen, omentum, kidney, mandibular LN**	−	169
6	2010	D	3	F	Born and raised in farm D	Weakness, mastitis, pneumonia, neurologic signs	Mammary gland **(milk), spleen**	−	169
7	2010	D	3	F	Born and raised in farm D	Weakness, bloated, constipation	**Lung, spleen**, kidney	Endocardial hemorrhage, abomasitis, pitting scar in liver	46
8	2010	D	3	F	Born and raised on farm D	Cough, hyperpnea	**Lung, spleen,** kidney	Hydropericardium, petechial hemorrhage of small intestine, multifocal necrosis of liver	169
9	2011	E	3	F	Bought from other farms	Chronic pneumonia, mastitis	Lung, liver, **spleen**, kidney	−	168
10	2012	F	3	M	Bought from other farms	Chronic pneumonia	**Lung**, liver, kidney	Myocardial and endocardial hemorrhage, congestion of pancreas and small intestine	1089

* LN = lymph node.

† Organs shown in bold were culture positive for *Burkholderia pseudomallei*.

‡ Genotyping was performed by using multilocus sequence typing (MLST) as described.^7^
